# A public health milestone: China publishes new Physical Activity and Sedentary Behaviour Guidelines

**DOI:** 10.1186/s44167-022-00009-x

**Published:** 2022-12-02

**Authors:** Sitong Chen, Jiani Ma, Jintao Hong, Cheng Chen, Yanxiang Yang, Zhen Yang, Peixuan Zheng, Yiling Tang

**Affiliations:** 1 Institute for Health and Sport, Victoria University, Melbourne, Australia; 2 Centre for Sport, Exercise and Health Sciences, Coventry University, Coventry, UK; 3 School of Health and Social Development, Deakin University, Geelong, Australia; 4 Shanghai Research Institute of Sports Science (Shanghai Anti-Doping Agency), Shanghai, China; 5 Institute of Sports and Sports Science, Karlsruhe Institute of Technology (KIT), Karlsruhe, Germany; 6 Chair of Sport and Health Management, Technical University of Munich, Munich, Germany; 7 Physical Activity, Sports & Health Research Group, Faculty of Movement and Rehabilitation Sciences, KU Leuven, Leuven, Belgium; 8 Department of Kinesiology, The University of Alabama, Tuscaloosa, USA; 9 School of Kinesiology, University of British Columbia, Vancouver, Canada

**Keywords:** Lifestyle, Health behaviour, Population health, Guideline, China

## Abstract

Physical inactivity has long been a global public health issue. In response to this, China published new Physical Activity and Sedentary Behaviour Guidelines for Chinese People in 2021 (PASBG 2021). This is a milestone in China’s public health, behavioural epidemiology and an important contribution to the *Healthy China 2030* initiative. This commentary summarises the contents and highlighted the significance of the new guidelines. The new Chinese PASBG provide foundations for population-based estimates of healthy behaviours, strategies addressing physical inactivity and messages designed to encourage people to be more active. While the contents of the PASBG 2021 are mostly consistent with the World Health Organisation physical activity guidelines, it is unclear on what evidence they are based, and whether this included research in Chinese people. Physical activity research in China is very limited and it is urgently needed to advance national-based physical activity research in China in accordance with the behavioural epidemiology framework. The development of new PASBG is only the first step, now it is what is done to communicate and disseminate, provide opportunities and supportive environments that will make a difference to physical activity levels in China. As such, we hope the PASBG 2021 will not only become a document for educating Chinese people to move more, but also an impetus for improving population health research.

## Background

The health benefits of sufficient physical activity (PA) and limited sedentary behaviour (SB) are well established [1–3]. The development of PA and SB guidelines is an important public health initiative because it can serve to promote active lifestyles at the populational level [4, 5]. As the prevalence of PA is low and the prevalence of SB is high [6, 7], many countries (e.g., Canada, the USA, Australia) as well as the World Health Organisation (WHO) have published PA and SB guidelines [1, 8–10]. The development of evidence-based PA and SB guidelines can inform the general public of the health benefits of PA and SB and guide in how to become more physically active and less sedentary.

Few studies have examined PA and SB levels in the People’s Republic of China. However, those studies that did examine this found generally very concerning levels of PA and SB across all age groups [11–15]. For example, based on the WHO guidelines, less than 13% of Chinese children and adolescents met the PA recommendations [16]. Furthermore, only 22.4% of adults adhered to the PA recommendations [13].

In response to this public health issue, PA promotion and SB reduction have been given greater attention by Chinese governments, researchers and communities. On December 29 2021, the Physical Activity and Sedentary Behaviour Guidelines (PASBG) for Chinese People 2021 (in Chinese: 中国人群身体活动指南2021) [17] was officially published by the Centres for Disease Control (CDC) of the National Health Commission of the People’s Republic of China. The PASBG for Chinese people aims to prevent noncommunicable diseases and foster a healthy and active society.

The development of the PAG was initialised in 2018 and led by the Guideline Development Advisory Commission (GDAC) [16]. In 2020, the GDAC systematically evaluated the scientific evidence regarding the health benefits of PA, interviewed and consulted over 50 relevant experts from China (e.g., in medicine and exercise), and summarised their comments and suggestions. This work resulted in research-driven and evidence-based guidelines.

## Guideline content

The Chinese PASBG mostly aligns with international guidelines (e.g., the World Health Organisation guidelines) [1, 9]. The guidelines consist of general principles and age-specific recommendations for children under 2 years old, children from 3 to 5 years old, children and adolescents from 6 to 17 years old, adults from 18 to 64 years old, older adults over 65 years old, and people living with chronic conditions. The Chinese PASBG comprises four general principles (see Table 1), which can be understood as follows: “moving is good, moving more is better, moving appropriately and moving regularly” [17]. This simple, concise and culturally relevant message is beneficial to the dissemination of the guidelines to the general public.

**Table 1:** Principles of the Physical Activity and Sedentary Behavior Guidelines for Chinese people

General principles in English	General principles in Chinese
1. Moving is good, moving more is better, moving appropriately and moving regularly	动则有益, 多动更好, 适度量力, 贵在坚持
2. Reduce sedentary behavior and keep physically active every day	减少静态行为, 每天保持身体活跃状态
3. Adhere to the recommended physical activity levels	身体活动达到推荐量
4. Participate in physical activity safely	安全地进行身体活动

Table 2 presents the Chinese PASBG for different age groups. Their contents are consistent with those of other guidelines [1, 2, 9, 18]. Interestingly, in comparison with China’s previous guidelines (published in 2011), several noteworthy differences should be mentioned. The 2011 guidelines only focused on adults, while the new guidelines target the entire life course. The 2011 guidelines only focused on PA, while the new guidelines also focus on SB, although only for children and adolescents. Unlike the previous guidelines, the new guidelines also focus on people living with disability, pregnant women and people living with cancer, type II diabetes or cardiovascular disease.

**Table 2:** Specific recommendations on physical activity and sedentary behavior for Chinese people

Population(s)	Children and adolescents			Adults	Older adults	People living with chronic conditions
	≤ 2 years	3–5 years	6–17 years	18–64 years	65 years and older	
Physical activity	(1) Daily interactive play with caregivers (2) ≥ 180 min of physical activity per day in independently mobile children	(1) ≥ 180 min of physical activity per day (2) ≥ 60 min of energetic play, and more outdoor activities	(1) ≥ 60 min of moderate to vigorous physical activity per day (2) Outdoor activities are highly encouraged (3) ≥ 3 days of muscle strengthening exercise a week	(1) ≥ 150–300 min of moderate intensity aerobic physical activity or 75–150 min of vigorous-intensity aerobic physical activity; or an equivalent combination of moderate and vigorous intensity activity throughout the week (2) ≥ 2 days of muscle strengthening exercise a week (3) Maintaining daily physical activity and increase the amount of physical activity (4) For women during pregnancy, moderate-intensity activities such as moderate-speed walking are encouraged every day, with a total of 150 min of moderate-intensity physical activity a week	(1) Recommendations for adults are applicable to older adults (2) Maintaining activities for coordination balance, agility, and flexibility (3) Those who cannot meet the recommend 150 min of moderate intensity physical activity should try to increase levels of physical activity	(1) People should consult with medical professionals prior to physical activity and do under professional guidance (2) If physically possible, people can adhere to age-specific the guidelines for general population (3) If physically possible, people should conditionally engage in regular physical activity (4) For patients with cardiocerebral vascular disease, breast cancer, colon cancer, prostate cancer, ovarian cancer, chronic obstructive pulmonary disease, and diabetes, appropriate physical activity is acceptable for all of them living with chronic conditions except during acute episodes
Sedentary behavior	(1) Not be restrained for more than 1 h at a time (2) Screen time is not recommended	(1) No more than 1 h per day of SB at a time (2) Screen time per day should be no more than 1 h	(1) No more than 1 h per day of sedentary behavior at a time (2) Screen time per day should be no more than 2 h	None	None	None

## Importance of the guidelines

The most important role of the Chinese PASBG is that the goal will be to educate the public on how to increase PA and reduce SB, as they recommend specific types, intensity, duration, and frequency of PA or SB for populations of different ages and those living with chronic disease. These messages are also important for clinical practice and for health care service professionals.

The Chinese PASBG will also provide a foundation for the surveillance and monitoring of PA and SB. Based on the new guidelines and recommended levels, researchers can assess populational levels of PA and active play in children, as well as moderate-to-vigorous PA and muscle-strengthening activities in adults and older adults. This will be useful in updating national health data and for the development of public health policy. Surveillance data are urgently needed, as little is known about the levels of PA and SB at the populational level in China [3, 12]. As the Chinese PASBG align with the guidelines of other countries and the WHO, the PA and SB levels of the Chinese population will be comparable to those of other countries.

The development of the new Chinese PASBG represents an important contribution to the global strategy for promoting active lifestyles. The WHO has launched the Global Action Plan on Physical Activity 2018–2030 (GAPPA) to combat the global prevalence of insufficient PA and excessive SB [19]. As such, WHO members are urged to make serious efforts to help achieve GAPPA’s ambitious goals. The development of the new PASBG demonstrates China’s commitment to GAPPA and continuous efforts to reduce the prevalence of physical inactivity among Chinese people. It also suggests that the Chinese government has an action plan to address the public health crisis of inactive and sedentary lifestyles [20–22].

Importantly, the key messages and four principles of the Chinese PASBG can be summarised in just 16 Chinese characters (动则有益, 多动更好, 适度量力, 贵在坚持). A simple, easy-to-understand and culturally relevant message is very powerful within a public health context and has the potential to achieve change.

While developing and issuing the PASBG is an important step in response to the *Healthy China 2030 Plan*, having guidelines itself will not change population PA and SB behaviours—it is what is done to communicate and disseminate, provide opportunities and supportive environments that will make a difference. As such, it is important that Chinese Governments, at all levels and across different departments, take the next step to actively implement and disseminate the PASBG in appropriate formats tailored to different demographics across China, as well as provide the conditions that will make it easier for people to be more active and less sedentary.

## Perspectives on future updates of the guidelines

While a large body of evidence has confirmed the beneficial impact of sufficient PA and limited SB on health, this evidence is mainly based on populations from Western countries [23]. Owing to cultural and genetic differences [24], it is possible that the health effects of PA and SB in Western populations cannot be fully replicated in the Chinese population [3]. As such, under ideal circumstances, the Chinese PASBG would be based on studies conducted in Chinese populations.

However, to date, we do not know on which evidence-based publications the PASBG are based. When examining the WHO [1], American [25], and Canadian [9, 26] guidelines, researchers can easily access scientific advisory reports that include the latest evidence and that form the basis for formulating the new guidelines. For example, web annex evidence profiles [23] and many research papers [1, 2, 18, 27–32] contain supportive evidence of the WHO Physical Activity and Sedentary Behaviour Guidelines. These complementary research-driven documents help professionals, policy-makers and the public better understand the contents of the published guidelines and confirm their high quality. As such, we hope that the GDAC will publish a scientific advisory report that supports the new PASBG. In addition, although the GDAC announced that they did conduct a vigorous and systematic scientific review, we are unaware of the process that was used in developing the PASBG. Ideally, future versions or updates of the PASBG will apply a more transparent development process and consider, for example, using the GRADE-ADOLOPMENT approach [33], which is a framework for the adoption, adaptation, and de-novo development of trustworthy recommendations.

Only people living with cancer, type II diabetes, and cardiovascular diseases are targeted in the Chinese guidelines [17]. This means that people living with other chronic conditions may be overlooked in clinical settings. However, accumulating evidence suggests that most people, even those living with specific conditions, can participate in appropriate PA [34]. As such, we hope that future editions of the PAG can provide specific recommendations for people living with other diseases, in line with information provided by the WHO guidelines (e.g., hypertension, HIV) [8].

Finally, while it is promising that the Chinese PASBG addresses limiting SB in children and adolescents, it is unclear why the PASBG does not include recommendations on SB for adults, which is inconsistent with the WHO [8] and Canadian guidelines [35]. The limited evidence available indicates that Chinese adults are exposed to prolonged SB [13], as such this gap should be addressed in the future, as SB has been recognised as an important health risk factor [36]. Furthermore, other countries, such as Canada, have developed 24-h Movement Guidelines that incorporate sleep [9, 10, 35]. A 24-h approach to movement behaviours should be considered in future versions of the PASBG.

## Future research needs

The development of the new Chinese PASBG is a welcome development and a milestone for public health. However, PA and SB research in China is still in its infancy. New research would strengthen the underpinnings of future guidelines. From the perspective of the behavioural epidemiology framework [37], more Chinese research is needed on (1) links between PA or SB and health outcomes, (2) methods for assessing PA and SB, (3) factors influencing PA and SB, (4) interventions to increase PA or decrease SB, and (5) translating intervention research into practice.

Research on the long-term health benefits of sufficient PA and limited SB in Chinese people is urgently needed, as the current evidence is mainly based on cross-sectional studies [3]. Among Chinese people, evidence remains scarce, especially from longitudinal studies and randomised controlled trials [3]. Additionally, little is known about the associations of different modalities of PA or different types of SB with health outcomes in different populations.

Surveillance and monitoring of PA and SB, alongside associated health outcomes, among Chinese people on a large scale are also required [3, 38]. Although some national data have been reported, most surveillance and monitoring systems are cross-sectional and use self-report measures; longitudinal and device-measured data are thus still lacking. Surveillance and monitoring of PA and SB in the general population is a current priority and should include people living with different health conditions as well as individuals of different ethnicities.

Research on PA and SB correlates and determinants in China is mainly focused on young people [39] and is very limited in any other age group. Again, most of this evidence is based on cross-sectional data. Moreover, many studies have focused on individual and interpersonal correlates and determinants [3, 39], but little is known about other factors (e.g., culture, policy) involved in PA and SB among Chinese people.

Owing to real-world complexity and cultural differences, interventions to increase PA or reduce SB in China face many challenges and are therefore very rare [3, 11]. Furthermore, the successful scale-up of effective interventions and translation of research evidence in China are also lacking. Likewise, China-based PA and SB policy research is extremely rare. This inhibits a comprehensive and insightful understanding of PA and SB policy in China.

It is unclear whether the Chinese PASBG can achieve the health impacts of promoting PA and decreasing SB. However, prior to achieving this aim, the first step is to broadly disseminate the guidelines, particularly to the general public, in addition to health practitioners. To make more people aware of the guidelines, government and non-government collaborative support is needed, using multi-dimensional approaches to integrate the guidelines into the workplace, school, community and family as well as other key settings. Enhancing people’s awareness and strengthening their knowledge of the Chinese guidelines is essential to achieve impact. Translating knowledge of the guidelines into practice is an important next step in promoting active lifestyles. As there is no standardised action plan to efficiently disseminate the guidelines for different populations (e.g., school-aged children, parents, employees), it is important to disseminate the guidelines using theory-based dissemination approaches [27] and to then examine the health impacts of adhering to the guidelines.

## Conclusions

The development and launch of the Physical Activity and Sedentary Behaviour Guidelines for Chinese People 2021 is a milestone in the history of public health in China and has the potential to contribute greatly to global health. However, strong efforts are needed to widely disseminate the guidelines across the large geographically and ethnically dispersed Chinese population. Moreover, the Chinese PASBG have several imitations that should be addressed in future versions. Finally, the Chinese PASBG should be underpinned by Chinese research on PA and SB, but there is a lack of such research across all stages of the behavioural epidemiology framework, and this urgently needs to be addressed.

## Data Availability

Data sharing is not applicable to this article as no datasets were generated or analysed during the current study.

## Abbreviations

PA: Physical activity
SB: Sedentary behaviour
WHO: World Health Organization
PASBG: Physical Activity and Sedentary Behaviour Guidelines
CDC: Centre for Disease Control
GDAC: Development Advisory Commission
GAPPA: Global Action Plan on Physical Activity

## References

[1] Bull FC, Al-Ansari SS, Biddle S, Borodulin K, Buman MP, Cardon G, Carty C, Chaput J-P, Chastin S, Chou R, et al. World Health Organization 2020 guidelines on physical activity and sedentary behaviour. Br J Sports Med. 2020;54(24):1451. 33239350 10.1136/bjsports-2020-102955 PMC7719906

[2] Chaput J-P, Willumsen J, Bull F, Chou R, Ekelund U, Firth J, Jago R, Ortega FB, Katzmarzyk PT. 2020 WHO guidelines on physical activity and sedentary behaviour for children and adolescents aged 5–17 years: summary of the evidence. Int J Behav Nutr Phys Act. 2020;17(1):141. 33239009 10.1186/s12966-020-01037-z PMC7691077

[3] Chen P, Wang D, Shen H, Yu L, Gao Q, Mao L, Jiang F, Luo Y, Xie M, Zhang Y, et al. Physical activity and health in Chinese children and adolescents: expert consensus statement (2020). Br J Sports Med. 2020;54(22):1321–31. 32471813 10.1136/bjsports-2020-102261 PMC7606574

[4] Ding D, Lawson KD, Kolbe-Alexander TL, Finkelstein EA, Katzmarzyk PT, van Mechelen W, Pratt M. The economic burden of physical inactivity: a global analysis of major non-communicable diseases. Lancet. 2016;388(10051):1311–24. 27475266 10.1016/S0140-6736(16)30383-X

[5] Lee IM, Shiroma EJ, Lobelo F, Puska P, Blair SN, Katzmarzyk PT. Effect of physical inactivity on major non-communicable diseases worldwide: an analysis of burden of disease and life expectancy. Lancet. 2012;380(9838):219–29. 22818936 10.1016/S0140-6736(12)61031-9 PMC3645500

[6] Aubert S, Barnes JD, Abdeta C, Abi Nader P, Adeniyi AF, Aguilar-Farias N, Andrade Tenesaca DS, Bhawra J, Brazo-Sayavera J, Cardon G, et al. Global matrix 3.0 physical activity report card grades for children and youth: results and analysis from 49 countries. J Phys Activity Health. 2018;15(s2):S251–73. 10.1123/jpah.2018-0472 30475137

[7] Guthold R, Stevens GA, Riley LM, Bull FC. Worldwide trends in insufficient physical activity from 2001 to 2016: a pooled analysis of 358 population-based surveys with 1·9 million participants. Lancet Glob Health. 2018;6(10):e1077–86. 30193830 10.1016/S2214-109X(18)30357-7

[8] Organization WH. WHO guidelines on physical activity and sedentary behaviour. Geneva: World Health Organization; 2020. 33369898

[9] Tremblay MS, Carson V, Chaput J-P. Introduction to the Canadian 24-Hour Movement Guidelines for Children and Youth: an integration of physical activity, sedentary behaviour, and sleep. Appl Physiol Nutr Metab. 2016;41(6 Suppl. 3):S311–27. 27306430 10.1139/apnm-2016-0203

[10] Ross R, Tremblay M. Introduction to the Canadian 24-Hour Movement Guidelines for Adults aged 18–64 years and adults aged 65 years or older: an integration of physical activity, sedentary behaviour, and sleep. Appl Physiol Nutr Metab. 2020;45(10 Suppl 2):S57–102. 33054330 10.1139/apnm-2020-0843

[11] Bao R, Chen S-T, Wang Y, Xu J, Wang L, Zou L, Cai Y. Sedentary behavior research in the Chinese population: a systematic scoping review. Int J Environ Res Public Health. 2020;17(10):3576. 32443711 10.3390/ijerph17103576 PMC7277100

[12] Shen H, Yan J, Hong J-T, Clark C, Yang X-N, Liu Y, Chen S-T. Prevalence of physical activity and sedentary behavior among Chinese children and adolescents: variations, gaps, and recommendations. Int J Environ Res Public Health. 2020;17(9):3066. 32354193 10.3390/ijerph17093066 PMC7246713

[13] Tian Y, Jiang C, Wang M, Cai R, Zhang Y, He Z, Wang H, Wu D, Wang F, Liu X, et al. BMI, leisure-time physical activity, and physical fitness in adults in China: results from a series of national surveys, 2000–14. Lancet Diabetes Endocrinol. 2016;4(6):487–97. 27133172 10.1016/S2213-8587(16)00081-4

[14] Gao Y, Cronin NJ, Nevala N, Finni T. Validity of long-term and short-term recall of occupational sitting time in Finnish and Chinese office workers. J Sport Health Sci. 2020;9(4):345–51. 32768127 10.1016/j.jshs.2017.06.003 PMC7411120

[15] Zhu Z, Tang Y, Zhuang J, Liu Y, Wu X, Cai Y, Wang L, Cao Z-B, Chen P. Physical activity, screen viewing time, and overweight/obesity among Chinese children and adolescents: an update from the 2017 physical activity and fitness in China—the youth study. BMC Public Health. 2019;19(1):197. 30767780 10.1186/s12889-019-6515-9 PMC6376726

[16] Chen S-T, Liu Y, Tremblay MS, Hong J-T, Tang Y, Cao Z-B, Zhuang J, Zhu Z, Wu X, Wang L, et al. Meeting 24-h movement guidelines: prevalence, correlates, and the relationships with overweight and obesity among Chinese children and adolescents. J Sport Health Sci. 2021;10(3):349–59. 32679341 10.1016/j.jshs.2020.07.002 PMC8167320

[17] Composing and Editorial Board of Physical Activity Guidelines for Chinese. Physical Activity Guidelines for Chinese (2021). Chinese Journal of Public Health, 2022, 38(2): 129–130. doi: 10.11847/zgggws1137503.

[18] Willumsen J, Bull F. Development of WHO guidelines on physical activity, sedentary behavior, and sleep for children less than 5 years of age. J Phys Act Health. 2020;17(1):96–100. 31877559 10.1123/jpah.2019-0457

[19] Organization WH. Global action plan on physical activity 2018–2030: more active people for a healthier world. Geneva: World Health Organization; 2018.

[20] Li F, Chen P. Addressing the public health concerns of physical inactivity, low levels of fitness, and unhealthy weight among Chinese school-aged children. J Sport Health Sci. 2017;6(4):379–80. 30356658 10.1016/j.jshs.2017.10.002 PMC6189249

[21] Li F, Mao L, Chen P. Physical activity and prevention of chronic disease in Chinese youth: a public health approach. J Sport Health Sci. 2019;8(6):512–5. 31720059 10.1016/j.jshs.2019.06.008 PMC6835010

[22] Li F. Physical activity and health in the presence of China’s economic growth: meeting the public health challenges of the aging population. J Sport Health Sci. 2016;5(3):258–69. 30356539 10.1016/j.jshs.2016.06.004 PMC6188738

[23] World Health O. WHO guidelines on physical activity and sedentary behaviour: web annex: evidence profiles. Geneva: World Health Organization; 2020.

[24] Chung HC, Keiller DR, Roberts JD, Gordon DA. Do exercise-associated genes explain phenotypic variance in the three components of fitness? A systematic review & meta-analysis. PLoS ONE. 2021;16(10): e0249501. 34648504 10.1371/journal.pone.0249501 PMC8516263

[25] Piercy KL, Troiano RP, Ballard RM, Carlson SA, Fulton JE, Galuska DA, George SM, Olson RD. The physical activity guidelines for Americans. JAMA J Am Med Assoc. 2018;320(19):2020–8. 10.1001/jama.2018.14854 PMC9582631 30418471

[26] Tremblay MS, Chaput J-P, Adamo KB, Aubert S, Barnes JD, Choquette L, Duggan M, Faulkner G, Goldfield GS, Gray CE, et al. Canadian 24-Hour Movement Guidelines for the early years (0–4 years): an integration of physical activity, sedentary behaviour, and sleep. BMC Public Health. 2017;17(5):874. 29219102 10.1186/s12889-017-4859-6 PMC5773896

[27] Tomasone JR, Kauffeldt KD, Morgan TL, Magor KW, Latimer-Cheung AE, Faulkner G, Ross-White A, Poitras V, Kho ME, Ross R. Dissemination and implementation of national physical activity, sedentary behaviour, and/or sleep guidelines among community-dwelling adults aged 18 years and older: a systematic scoping review and suggestions for future reporting and research. Appl Physiol Nutr Metab. 2020;45(10 Suppl. 2):S258–83. 33054340 10.1139/apnm-2020-0251

[28] Hämäläinen R-M, Breda J, da Silva GF, Gongal G, Khan W, Mendes R, Nederveen L, Ramanandraibe N, Sako B, Whiting S. New global physical activity guidelines for a more active and healthier world: the WHO Regional Offices perspective. Br J Sports Med. 2020;54(24):1449. 33239349 10.1136/bjsports-2020-103531

[29] Milton K, Bauman AE, Faulkner G, Hastings G, Bellew W, Williamson C, Kelly P. Maximising the impact of global and national physical activity guidelines: the critical role of communication strategies. Br J Sports Med. 2020;54(24):1463. 33239351 10.1136/bjsports-2020-102324 PMC7719904

[30] Dempsey PC, Biddle SJH, Buman MP, Chastin S, Ekelund U, Friedenreich CM, Katzmarzyk PT, Leitzmann MF, Stamatakis E, van der Ploeg HP, et al. New global guidelines on sedentary behaviour and health for adults: broadening the behavioural targets. Int J Behav Nutr Phys Act. 2020;17(1):151. 33239026 10.1186/s12966-020-01044-0 PMC7691115

[31] DiPietro L, Al-Ansari SS, Biddle SJH, Borodulin K, Bull FC, Buman MP, Cardon G, Carty C, Chaput J-P, Chastin S, et al. Advancing the global physical activity agenda: recommendations for future research by the 2020 WHO physical activity and sedentary behavior guidelines development group. Int J Behav Nutr Phys Act. 2020;17(1):143. 33239105 10.1186/s12966-020-01042-2 PMC7690200

[32] Segar ML, Marques MM, Palmeira AL, Okely AD. Everything counts in sending the right message: science-based messaging implications from the 2020 WHO guidelines on physical activity and sedentary behaviour. Int J Behav Nutr Phys Act. 2020;17(1):135. 33148305 10.1186/s12966-020-01048-w PMC7643438

[33] Schünemann HJ, Wiercioch W, Brozek J, Etxeandia-Ikobaltzeta I, Mustafa RA, Manja V, Brignardello-Petersen R, Neumann I, Falavigna M, Alhazzani W, et al. GRADE Evidence to Decision (EtD) frameworks for adoption, adaptation, and de novo development of trustworthy recommendations: GRADE-ADOLOPMENT. J Clin Epidemiol. 2017;81:101–10. 27713072 10.1016/j.jclinepi.2016.09.009

[34] Dempsey PC, Friedenreich CM, Leitzmann MF, Buman MP, Lambert E, Willumsen J, Bull F. Global Public Health Guidelines on physical activity and sedentary behavior for people living with chronic conditions: a call to action. J Phys Act Health. 2020;1:76–85. 10.1123/jpah.2020-0525 33276323

[35] Ross R, Chaput J-P, Giangregorio LM, Janssen I, Saunders TJ, Kho ME, Poitras VJ, Tomasone JR, El-Kotob R, McLaughlin EC, et al. Canadian 24-Hour Movement Guidelines for adults aged 18–64 years and adults aged 65 years or older: an integration of physical activity, sedentary behaviour, and sleep. Appl Physiol Nutr Metab. 2020;45(10 Suppl. 2):S57–102. 33054332 10.1139/apnm-2020-0467

[36] Saunders TJ, McIsaac T, Douillette K, Gaulton N, Hunter S, Rhodes RE, Prince SA, Carson V, Chaput JP, Chastin S, et al. Sedentary behaviour and health in adults: an overview of systematic reviews. Appl Physiol Nutr Metab. 2020;45(10 Suppl. 2):S197-s217. 33054341 10.1139/apnm-2020-0272

[37] Sallis JF, Owen N, Fotheringham MJ. Behavioral epidemiology: a systematic framework to classify phases of research on health promotion and disease prevention. Ann Behav Med. 2000;22(4):294–8. 11253440 10.1007/BF02895665

[38] Liu Y. Promoting physical activity among Chinese youth: no time to wait. J Sport Health Sci. 2017;6(2):248–9. 30356574 10.1016/j.jshs.2017.03.014 PMC6189231

[39] Lu C, Stolk RP, Sauer PJJ, Sijtsma A, Wiersma R, Huang G, Corpeleijn E. Factors of physical activity among Chinese children and adolescents: a systematic review. Int J Behav Nutr Phys Act. 2017;14(1):36. 28320408 10.1186/s12966-017-0486-y PMC5360041

